# Bone Marrow-Derived Mesenchymal Stem Cells Maintain the Resting Phenotype of Microglia and Inhibit Microglial Activation

**DOI:** 10.1371/journal.pone.0084116

**Published:** 2013-12-31

**Authors:** Ke Yan, Run Zhang, Chengmei Sun, Lei Chen, Peng Li, Yi Liu, Lingmei Peng, Haitao Sun, Kun Qin, Fanfan Chen, Weiyi Huang, Yuxin Chen, Bingke Lv, Mouxuan Du, Yuxi Zou, Yingqian Cai, Lingsha Qin, Yanping Tang, Xiaodan Jiang

**Affiliations:** 1 The National Key Clinic Specialty, The Neurosurgery Institute of Guangdong Province, Guangdong Provincial Key Laboratory on Brain Function Repair and Regeneration, Department of Neurosurgery, Zhujiang Hospital, Southern Medical University, Guangzhou, China; 2 Department of Neurosurgery, Shenzhen Second People's Hospital, the First Affiliated Hospital of Shenzhen University, Shenzhen, China; 3 Department of Neurosurgery, Guangzhou First People's Hospital, Guangzhou, China; 4 Department of Neurology, The First People's Hospital of Foshan, Foshan, China; 5 Department of Neurosurgery, Guangdong General Hospital, Guangdong Academy of Medical Science, Guangzhou, China; University of Nebraska Medical Center, United States of America

## Abstract

Many studies have shown that microglia in the activated state may be neurotoxic. It has been proven that uncontrolled or over-activated microglia play an important role in many neurodegenerative disorders. Bone marrow-derived mesenchymal stem cells (BMSCs) have been shown in many animal models to have a therapeutic effect on neural damage. Such a therapeutic effect is attributed to the fact that BMSCs have the ability to differentiate into neurons and to produce trophic factors, but there is little information available in the literature concerning whether BMSCs play a therapeutic role by affecting microglial activity. In this study, we triggered an inflammatory response situation *in vitro* by stimulating microglia with the bacterial endotoxin lipopolysaccharide (LPS), and then culturing these microglia with BMSC-conditioned medium (BMSC-CM). We found that BMSC-CM significantly inhibited proliferation and secretion of pro-inflammatory factors by activated microglia. Furthermore, we found that the phagocytic capacity of microglia was also inhibited by BMSC-CM. Finally, we investigated whether the induction of apoptosis and the production of nitric oxide (NO) were involved in the inhibition of microglial activation. We found that BMSC-CM significantly induced apoptosis of microglia, while no apoptosis was apparent in the LPS-stimulated microglia. Our study also provides evidence that NO participates in the inhibitory effect of BMSCs. Our experimental results provide evidence that BMSCs have the ability to maintain the resting phenotype of microglia or to control microglial activation through their production of several factors, indicating that BMSCs could be a promising therapeutic tool for treatment of diseases associated with microglial activation.

## Introduction

As the resident immune cells of the central nervous system (CNS), microglia primarily participate in tissue defense and protection of the brain. They are deeply involved in lesions, stroke, brain tumors, and neurodegenerative diseases [1], and play an important role in antigen-presenting, phagocytosis of pathogens, cytokine production and nerve repair. In the normal brain, microglia are in a “resting” state, expressing low levels of most immune receptors such as pattern recognition receptors, chemokine receptors, and major histocompatibility complex molecules, which are all essential to the initiation and propagation of immune responses [2]. Numerous studies have provided evidence that microglia can be mobilized in response to many injuries and diseases of the CNS [3]–[5]. Threats to CNS homeostasis can transform microglia from a resting state to an activated state and cause them to undergo morphological and functional transformations. Activated microglia release more pro-inflammatory factors such as tumor necrosis factor (TNF)-α, interleukin (IL)-1β and nitric oxide (NO), which are neurotoxic [6], [7]. Furthermore, activated microglia show enhanced phagocytic activity, in which state they can phagocytose apoptotic neural cells, and even normal neurons [8]. Experiments have also shown that microglial activation is amplified and prolonged in the aged brain compared to the adult brain [9]. All this evidence demonstrates that microglia can have deleterious effects under some special circumstances, and that uncontrolled inflammatory responses caused by activated microglia contribute to the severity of traumatic brain injury (TBI) and many neurodegenerative diseases [6], [10]. There is also some evidence showing that blockade of microglial activation by anti-inflammatory agents such as minocycline attenuates pathology in Parkinson's disease [11].

Recently, mesenchymal stem cells (MSCs) have been considered as a promising donor source for tissue repair and regeneration [12]–[14]. These cells can be isolated from many adult tissues, including bone marrow, adipose tissue, placenta, and amniotic fluid [15]. It has been demonstrated that MSCs are multipotent and have the ability to differentiate into a variety of mesodermal lineages, including adipocytes, osteocytes and chondrocytes, as well as other embryonic lineages [16]. Because of their lack of immunogenicity, MSCs are able to escape the recognition of alloreactive T cells and natural killer cells. The therapeutic effect of MSCs has been proven in many studies. For example, MSCs have been used successfully in humans to control severe acute graft-versus-host disease (GVHD) of the gut and liver [12]. MSCs transplanted into the heart have the ability to promote cardiac tissue regeneration after myocardial infarction [13]. In vitro, MSCs can inhibit pancreatic islet antigen-specific T cell activation, providing evidence that MSCs may be beneficial for islet engraftment in type 1 diabetes [14]. All of these studies indicate that MSCs may be a promising tool for use in clinical therapy.

There is increasing evidence in animal models of traumatic brain injury (TBI) and spinal cord injury (SCI) that MSCs play an important role in the repair of central nervous system damage [17], [18]. The mechanism responsible for this phenomenon may be attributed to their transdifferentiation, enabling them to replace damaged neural cells and produce growth factors [19]. Many experiments have shown that MSCs transplanted into the brain or spinal cord can directionally migrate into the damaged tissue, and there differentiate into neuron-like cells expressing NeuN and into astrocytes expressing GFAP [20]. Furthermore, MSCs can produce an array of growth factors such as vascular endothelial growth factor (VEGF), brain-derived neurotrophic factor (BDNF), glia-derived neurotrophic factor (GDNF) and hepatocyte growth factor (HGF), all of which are crucial components for neural damage repair [21]–[24]. In spite of this, few studies have focused on whether MSCs exert their therapeutic role by affecting the activity of microglial cells. In this study, we used rat bone marrow-derived mesenchymal stem cells (BMSCs) in combination with allogeneic microglia, to investigate whether BMSCs have any influence on microglial activity. Additionally, we analyzed the possible mechanisms responsible for these effects.

## Methods

### Ethics statements

Sprague-Dawley (SD) rats were supplied by the Animal Experiment Center of Southern Medical University (Guangzhou, China). Animals were housed under a 12-h light/dark cycle, with food and water freely available. All of the experimental procedures were approved by the Southern Medical University Ethics Committee. All surgery was performed under sodium pentobarbital anesthesia, and efforts were made to minimize numbers and suffering of animals used.

### Generation of rat bone marrow-derived MSCs and collection of conditioned medium

BMSCs were obtained from adult male Sprague-Dawley rats weighing 80–120 g. Briefly, The femurs and tibias of the rats were dissected out under aseptic conditions, and then both ends of the bones were cut off. The cells were rinsed from the marrow cavity of the femurs and tibias with 5 mL of Dulbecco's Modified Eagle Medium/Nutrient Mixture F-12 (DMEM/F-12) using a 25-guage needle. The cells obtained were suspended in D-MEM/F-12 supplemented with 10% fetal bovine serum (FBS) and plated into 25cm^2^ culture flasks. After incubating for 3 days at 37°C in a 5% CO_2_ humidified atmosphere, the medium was changed in order to remove non-adherent cells. The remaining adherent cells (primary BMSCs) were passaged every 2 days. Cells from 3 to 5 generations were used in our experiments. The expression of the typical markers CD44 and CD90, and the absence of the hematopoietic markers CD34 and CD45, were assessed by cytofluorometric analysis.

BMSCs (5×10^5^ in 5 mL) were plated into 25 cm^2^ culture flasks in Dulbecco's Modified Eagle Medium/Nutrient Mixture F-12 (DMEM/F-12) containing 10% FBS. After 24 hours, the medium was replaced with 5 mL DMEM/F-12 without FBS and the cells were incubated for another 24 hours, at the end of which time, the medium was collected and centrifuged at 300× g for 3 minutes to remove debris before use. The medium thus obtained was defined as BMSC-conditioned medium (BMSC-CM) in our study.

### Microglia culture

Primary microglial cells were enriched in vitro using the shaking method described by Giulian and Baker [25]. Briefly, 2-day-old male SD rat were sacrificed and soaked in 75% ethanol for 1 minute. Cerebral hemispheres were dissected out following standard techniques and anatomical landmarks and then meninges were peeled off. The hippocampus, basal ganglion, and olfactory bulb were carefully removed with microsurgical instruments under a microscope, and the remaining cortical tissue was minced with a pair of microsurgical scissors. The shredded tissue was then incubated with 1 mL trypsin and 1 mL PBS for 15 min in a 37°C water bath with occasional swirling. After centrifuging at 300× g for 3 minutes, the cells were plated into 75 cm^2^ flasks which had been coated with poly-L-lysine. Mixed glial cells were cultured in DMEM/F-12 containing 10% FBS at 37°C in 5% CO_2_ in air and 95% humidity. The culture medium was replaced with 15 mL fresh growth medium after 24 hours. Subsequently, one-half of the volume of culture medium was replaced with an equal volume of fresh growth medium twice per week. The mixed glial cell cultures were incubated for 10–14 days. At the end of this period, stratification had been reached and the microglial cells in the upper layer could be harvested. Expression of the typical marker CD11b was assessed by cytofluorometric analysis.

### Treatments

The microglial cells were mainly divided into four experimental groups:

1) Control group: cells were incubated in DMEM/F-12 containing 10% fetal bovine serum (FBS).

2) LPS-treated group: cells were incubated in DMEM/F-12 containing 10% FBS and 1 µg/mL lipopolysaccharide (LPS).

3) Conditioned medium (CM) group: cells were incubated in BMSC-conditioned medium (described above) containing 10% FBS.

4) CM+LPS group: cells were incubated in BMSC-conditioned medium containing 10% FBS and 1 µg/mL lipopolysaccharide.

### Proliferation assays

Cell proliferation was measured at the indicated time-points using the Cell Counting Kit-8 assay (CCK-8, Dojindo, Japan). Briefly, after isolation from mixed glial culture, microglial cells were plated into 96-well plates at a density of 1×10^4^ cells/well with 100 µL of complete culture medium. After adhesion for 24 hours, the medium was changed to DMEM/F-12 or conditioned medium with or without LPS (1 µg/mL) and incubated for 12, 24, 48 and 72 hours. At the end of each culture period, 10 µL CCK8 reagent was added to each well and incubated for another 4 hours, at the end of which the absorbance was measured at a wavelength of 450 nm. In order to investigate whether the inhibitory effect of BMSCs was associated with NO, S-methylisothiourea sulfate (inhibitor of iNOS, 1 mM) was added to some groups to inhibit the secretion of NO from BMSCs. The conditioned medium derived from these groups was regarded as conditioned medium containing reduced NO. All of the experiments above were repeated at least three times.

### Cytokine and Chemokine Measurements

We used Bio-Plex Pro Assays to measure the cytokines, chemokines and growth factors secreted by microglial cells and bone marrow-derived mesenchymal stem cells. Briefly, 3 mL microglial cells were plated into 6-well plates at a density of 2×10^5^ cells/mL. These cells were divided into four treatment groups and cultured under the conditions described above. After incubating at 37°C for 48 hours, the supernatant was collected and centrifuged to remove debris. TNF-α, IL-1β, IL-4, IL-6, IL-10, IL-17, INF-γ, RANTES, MCP-1, MIP-2 and VEGF were measured by Bio-Plex Pro Assays (Bio-Rad Laboratories, Hercules, CA) in accordance with the manufacturer's instructions. Data analysis was performed using Bio-Plex Manager software.

### Phenotype analysis

We investigated whether BMSCs affect the expression of CD11b and CD68 by resting or activated microglia. After culturing the microglia in the four different treatment media for 48 hours, microglia in the four groups were digested with trypsin, washed three times with PBS, and kept at 4°C. Cells were then stained with antibodies against CD11b (FITC-labeled; BD, San Diego, CA) and CD68 (FITC-labeled; AbD Serotec, Oxford, UK). As a control, cells were stained with mouse IgG1 isotype-control antibodies. The changes in CD11b and CD68 were assessed by cytofluorometric analysis and the results were expressed as the percentage of positively stained cells relative to the total cell number.

### Phagocytosis assays

We incubated microglia with fluorescence-labeled latex beads to study their phagocytic activity. Briefly, 500 µL microglial cells were plated into 24-well plates at a density of 2×10^5^ cells/mL. Cells were cultured in DMEM/F-12 or conditioned medium with or without LPS (1 µg/mL) and incubated at 37°C in 5% CO_2_ in air and 95% humidity. After 24 hours, 5 µL fluorescence-labeled latex beads (L3030, Sigma) were added into each well and cultures were incubated for a further 4 hours. At the end of the incubation period the cells were fixed with 4% paraformaldehyde for 10 minutes, and then washed with PBS five times to remove serum and unbound beads. The cells were then stained with 4′,6-diamidino-2-phenylindole (DAPI) and observed under a fluorescence microscope to observe whether the microglia were able to phagocytose the beads. Phagocytic activity was illustrated by the number of beads phagocytosed by the cells.

### Quantification of apoptosis by TUNEL assay

The TUNEL assay, which was used for the detection of apoptosis, was performed using an *in situ* cell death detection kit, POD (Roche, Indianapolis, IN) in accordance with the manufacturer's instructions. Freshly-isolated microglial cells were seeded at 500 µL/well into 48-well plates at a density of 1×10^5^ cells/mL. Cells were incubated in DMEM/F-12 or conditioned medium with or without LPS (1 µg/mL) for 48 hours, following which cells were fixed with 4% paraformaldehyde for 20 minutes and permeabilized with 0.1% Triton X-100 for 10 minutes at room temperature. Subsequently, the cells were incubated with TUNEL reaction mixture (45 µL of label solution and 5 µL of enzyme solution) for 60 minutes at 37°C. Apoptotic nuclei, with green fluorescence, were observed under a fluorescence microscope at 400× magnification. The result was estimated as the percentage of TUNEL-positive cells among the total number of cells. The TUNEL reagent can also use streptavidin-horseradish peroxidase conjugate and diaminobenzidine (DAB) to detect apoptotic nuclei.

### Annexin V/propidium iodide assay

Apoptosis was also evaluated using annexin V/propidium iodide (PI) staining followed by flow cytometry. After treating with different media for 48 hours, microglial cells collected from the four groups were centrifuged at 300× g for 5 minutes and washed twice with cold PBS. Then the cells were resuspended in binding buffer and incubated with propidium iodide (PI) and annexin V-FITC (BD Biosciences Pharmingen, San Diego, CA) for 15 minutes at room temperature. A total of at least 10,000 events were collected and analyzed by flow cytometry (BD).

### Statistical analysis

All data are presented as mean ± SD. Comparisons between multiple groups were performed using one-way ANOVA followed by Student's unpaired t-test. A value of *P*<0.05 was considered statistically significant. All calculations were performed using the Statistical Package for the Social Sciences, version 13.0 (SPSS, Chicago, IL).

## Results

### BMSCs inhibit proliferation of microglia

**Figure 1:**
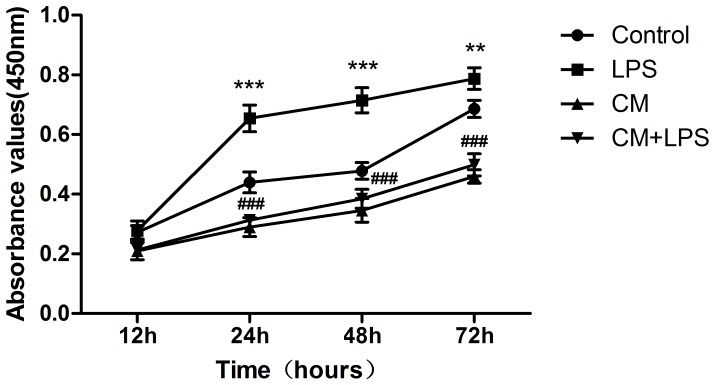
BMSCs inhibit proliferation of microglial cells. To determine whether BMSCs influence the growth of microglia, we used conditioned medium derived from BMSCs to culture microglia with or without LPS. The results show that LPS (1 µg/mL) promotes the proliferation of microglia (LPS groups vs. control groups, **P<0.01, and ***P<0.001), while BMSC-CM was effective in inhibiting the proliferation of microglia. The number of cells in the group treated with conditioned medium and LPS (1 µg/mL) decreased significantly compared with cells treated with LPS alone (CM+LPS group vs. LPS group, ###P<0.001).

**Figure 2:**
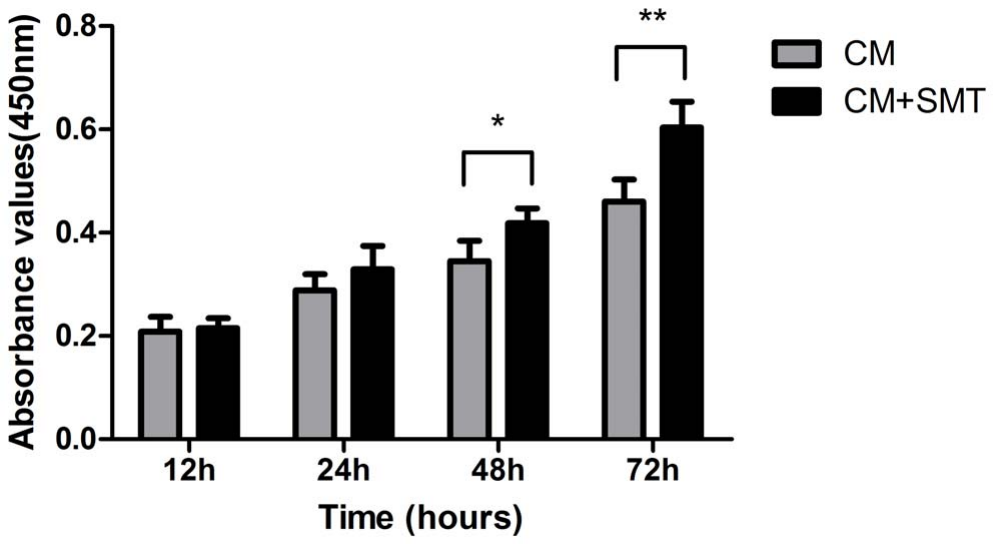
Role of NO in the BMSC-mediated inhibition of microglial proliferation. We cultured microglia in conditioned medium derived from BMSCs with or without inhibition of NO production. In the CM+SMT groups, microglia were incubated in conditioned medium derived from BMSCs in which NO production was inhibited by the NO inhibitor S-methylisothiourea sulfate (SMT, 1 mM). We found that cells cultured in CM after inhibition of NO production (CM+SMT groups) showed increased proliferation compared to cells cultured in CM without inhibition of NO production (CM groups). Bars represent means plus or minus SD obtained from six independent experiments. A P value of less than 0.05 (*), or less than 0.01 (**) was considered statistically significant.

We investigated whether BMSCs inhibit the proliferation of microglia, the innate immune cells of the CNS. The results of the CCK8 assays clearly show that proliferation of microglial cells was significantly increased in the LPS-stimulated group at the indicated time-points (LPS groups vs. control groups, *P*<0.001 at 24, 48 hours, and *P*<0.01 at 72 hours, Figure 1), while treatment with BMSC-CM significantly inhibited proliferation (CM groups vs. Control groups, *P*<0.001 at 24, 48 and 72 hours). The proliferation of cells treated with BMSC-CM after LPS stimulation decreased significantly compared with that of cells treated with LPS alone (CM+LPS group vs. LPS group, *P*<0.001 at 24, 48 and 72 hours). This phenomenon demonstrates that BMSCs inhibit the activity of activated (LPS-treated) microglia by secreting soluble factors. We further investigated whether the inhibitory effect of BMSCs was associated with NO by using S-methylisothiourea sulfate (an inhibitor of iNOS) to inhibit NO secretion, and found that cells cultured in this conditioned medium grew better than cells cultured in conditioned medium without the NO inhibitor (Figure 2).

### BMSCs inhibit phagocytosis by microglia

**Figure 3:**
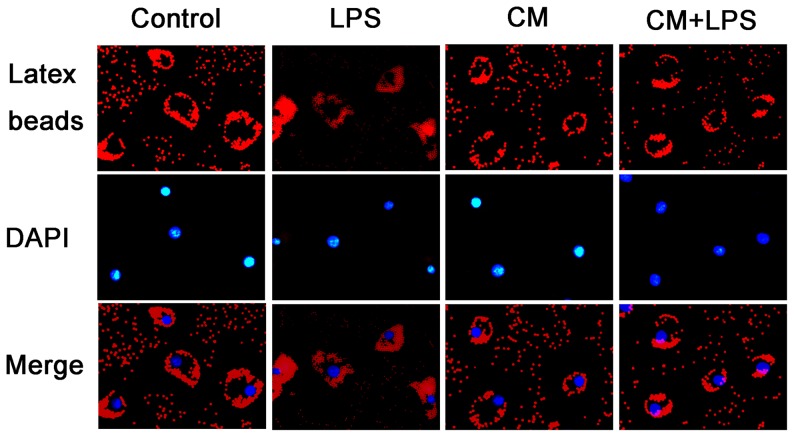
BMSCs inhibit phagocytosis by microglial cells. We tested the capacity of microglia to phagocytose fluorescent-labeled latex beads by observing them under a fluorescence microscope (magnification 400×). The microglial cells were incubated in DMEM/F-12 or conditioned medium with or without LPS (1 µg/mL) at 37°C in 5% CO2 in air and 95% humidity. The nuclei were counterstained with DAPI. Following incubation with LPS (1 µg/mL) for 4 hours, microglia clearly ingested more latex beads than untreated control groups, while the phagocytic activity of microglia in the conditioned medium (CM) treated-groups was significantly inhibited.

We used fluorescence microscopy to observe the phagocytosis of latex beads by microglia. As shown in Figure 3, we found that red fluorescent latex beads were phagocytosed most by microglia stimulated with LPS. Meanwhile, phagocytosis by microglia treated with BMSC-CM was significantly inhibited compared with the control groups. Activated microglia cultured in BMSC-CM exhibited reduced phagocytic capacity compared to cells cultured in LPS alone.

### BMSCs inhibit cytokine and chemokine secretion by microglia

**Figure 4:**
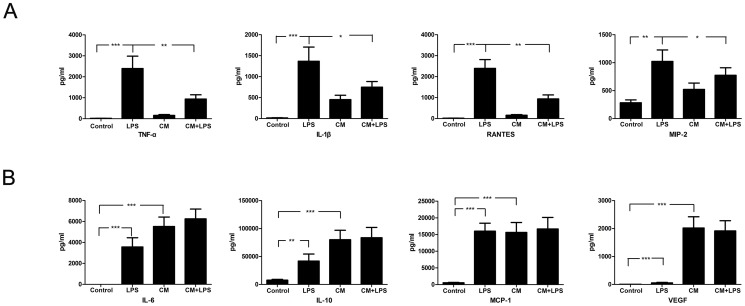
BMSCs inhibit cytokine and chemokine production by activated microglia. We analyzed cytokine and chemokine production by microglia using Bio-Plex Pro assays. (A) LPS-treated microglia produce more TNF-α, IL-1β, RANTES and MIP-2 than control microglia, while treatment with conditioned medium significantly inhibited production of these factors. (B) Microglia in the LPS-treated groups produced more IL-6, IL-10 and MCP-1 compared to control groups. The supernatant derived from conditioned medium-treated groups contained more IL-6, IL-10, MCP-1 and VEGF compared to control groups. A P value of less than 0.05 (*), less than 0.01 (**), and less than 0.001 (***) was considered statistically significant.

In addition to phagocytic activity, cytokine production is another important function of microglia. Therefore, in order to better characterize the inhibitory effect of BMSCs, we analyzed cytokine production by microglia cultured in BMSC-CM. Microglia have been shown to release many cytokines, chemokines, and free radicals under inflammatory conditions. After subjecting the cells to the different treatments used in our experiment, we collected the supernatants and analyzed cytokine levels using Bio-Plex Pro assays for TNF-α, IL-1β, IL-4, IL-6, IL-10, IL-17, INF-γ, RANTES, MCP-1, MIP-2 and VEGF. Our results show that in the presence of LPS, microglia secrete more pro-inflammatory cytokines such as TNF-α and IL-1β, and chemokines such as RANTES and MIP-2 than controls; however, BMSC-CM significantly inhibited secretion of these factors (Figure 4A). LPS-treated microglia also produced more anti-inflammatory cytokines such as IL-6 and IL-10, and chemokines such MCP-1 in comparison to the control groups (Figure 4B). We analyzed the differences between the CM-treated groups and the control groups, and found that the supernatant derived from CM-treated groups contained more IL-6, IL-10, MCP-1, and VEGF compared to control groups, indicating that BMSCs produce large quantities of these factors (Figure 4B). In contrast, virtually no IL-4, INF-γ, or IL-17 secretion was detected in the above four groups.

### BMSCs inhibit the LPS-induced expression of activated microglia receptors

**Figure 5:**
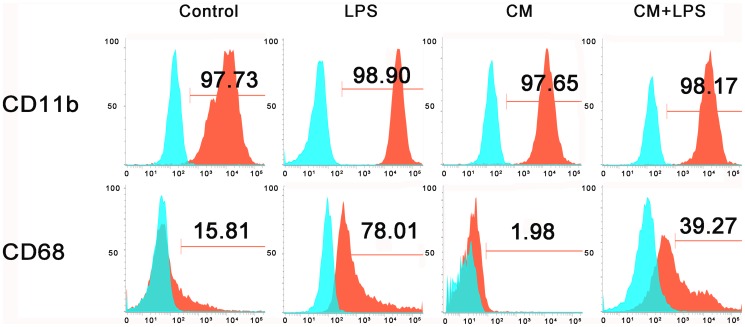
BMSCs alter LPS–induced upregulation of CD68 on microglia. Expression of CD11b and CD68 on microglia were assessed by cytofluorometric analysis. Cells were treated with DMEM/F-12 or conditioned medium with or without LPS and incubated for 48 hours. Red profiles represent expression of activating receptors; Blue profiles represent negative controls. Numbers indicate percentages of positive cells.

To investigate whether BMSCs could affect the surface expression of resting or activated microglia, the microglial phenotype was assessed by cytofluorometric analysis. As shown in Figure 5, after culture in LPS for 48 hours, cells displayed increased expression of CD68, a marker of activated microglia, whereas CD11b was not significantly modified. The expression of CD68 was significantly lower in microglia cultured in BMSC-CM compared to microglia cultured in DMEM/F-12, however, no downregulation of CD11b was detected. In the LPS+CM groups, cells showed lower expression of CD68 compared with cells in the LPS-treated groups, which indicated that BMSCs inhibit the LPS-induced expression of activated microglia receptors.

### BMSCs mediate microglial apoptosis

**Figure 6:**
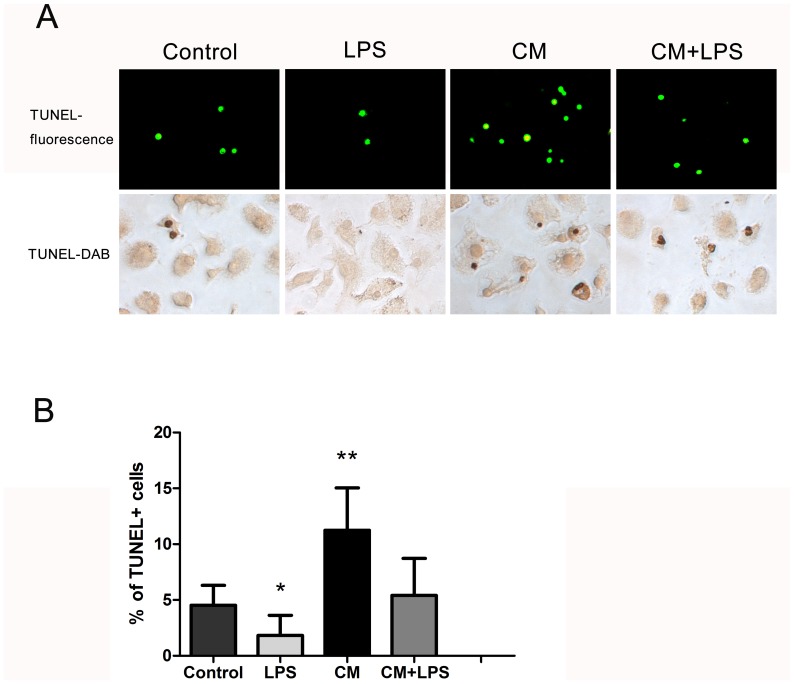
BMSC-induced apoptosis of microglial cells. We investigated the effects of conditioned medium on apoptosis of microglial cells by TUNEL assay. (A) Cells were observed under a fluorescence microscope (magnification 400×), or stained with DAB and observed under an optical microscope (magnification 400×). We found fewer apoptotic cells in the LPS-treated groups compared with the control groups, while apoptosis was increased in the groups cultured in BMSC-CM. (B) The percentage of apoptotic microglial cells among the total number of microglial cells in each group is shown. Bars represent means plus or minus SD obtained from eight random fields. *P<0.05, **P<0.01, compared with control groups.

**Figure 7:**
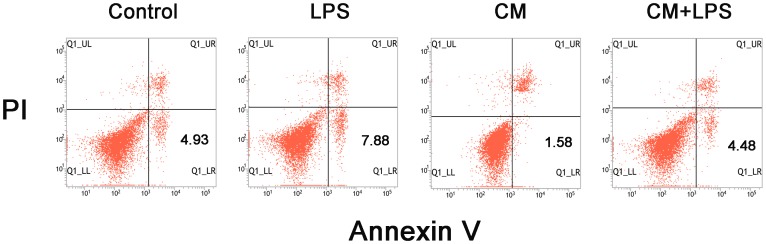
Apoptosis assessed by annexin V/propidium iodide (PI) assay. Annexin V/PI staining was performed to assess the apoptosis rate in microglia. After incubating in DMEM/F-12 or BMSC-CM with or without LPS (1 µg/mL) for 48 hours, microglia were harvested. Cells were then stained with annexin V-FITC and propidium iodide and analyzed by flow cytometry. The results are presented as density plots of propidium iodide vs. annexin V-FITC. Apoptotic cells have high annexin V-FITC and low propidium iodide staining (lower-right quadrant). Numbers on the graph represent the percentages of apoptotic cells in the different groups.

We further investigated the effects of BMSC-CM on apoptosis of microglial cells using the TUNEL assay. Apoptotic cells exhibited pyknotic or split nuclei which could be identified by green fluorescent staining (Figure 6A). We were surprised to find that treatment of the microglial cells with LPS (1 µg/mL) for 48 h reduced the number of apoptotic cells from 4.5% to 1.8% of total cells (Figure 6B), a significant reduction compared to the control groups (*P*<0.05). However, treatment with conditioned medium increased the percentage of apoptotic cells to 11.2% (*P*<0.01). The percentage of apoptotic cells was also increased in the group treated with both LPS and BMSC-CM compared to the group treated with LPS alone (*P*<0.05). Apoptosis assessed by annexin V/propidium iodide (PI) assay showed the same trends as the TUNEL assay. As shown in Figure 7 (a representative experiment), after exposure to different media for 48 hours, the percentage of apoptotic cells in the LPS-treated groups was reduced compared with the control groups, while apoptotic cells in the CM groups increased dramatically compared with the control groups. BMSC-CM also induced apoptosis of activated microglial cells, as the CM+LPS groups contained more apoptotic cells than the LPS-treated groups. These results indicate that BMSC-CM not only exerts an inhibitory effect on proliferation of microglia, but also promotes their apoptosis.

## Discussion

In our present study, we provide evidence that BMSCs maintain the resting phenotype of microglia and inhibit their activity in vitro. Following incubation in BMSC-CM for different time periods, microglia treated with or without LPS showed reduced proliferation, weakened phagocytic capacity, and decreased expression of CD68. The results of Bio-Plex Pro assays show that production of TNF-α, IL-1β, RANTES, and MIP-2 by microglia increased significantly after LPS stimulation, but was significantly inhibited by BMSC-CM. BMSC-CM also inhibits the production of these factors by activated microglia which have been treated with LPS. We further analyzed differences between supernatant from CM-treated groups and control groups, and found that supernatant from the CM-treated groups contained more IL-6, IL-10, MCP-1, and VEGF compared to control groups. Finally, we measured levels of IL-4, IFN-γ, and IL-17 in the supernatant from the four groups, and found that virtually no IL-4, IFN-γ or IL-17 was produced by microglia treated with BMSC-CM. We further investigated possible reasons for the inhibitory effect of BMSCs, and concluded that BMSC-induced apoptosis and NO secretion could be relevant factors.

There have been previous reports of the immunosuppressive capacity of MSCs. For example, MSCs inhibit the proliferation of lymphocytes [26]–[29] and dendritic cells [30], [31]. In recent years, MSCs have been commonly used by researchers to attenuate immunological rejection and thus prolong graft survival [32]. Our study shows that BMSCs inhibit the proliferation of microglia, the resident immune cells of the CNS. Microglia are generally considered to be able to clear apoptotic cells without causing inflammation under normal conditions [33], but in the case of traumatic brain injury, or of some neurodegenerative diseases such as Parkinson's disease and Alzheimer's disease, microglia are activated and can produce a lot of factors that are toxic to neighboring neurons. Since microglia are the most potent cells in initiating the CNS immune response, we believe that in order to identify the therapeutic mechanism of mesenchymal stem cells, it is important to figure out whether BMSCs influence the activity of microglia. In this study, we simulated possible influences of BMSCs in vitro by culturing microglia in BMSC-CM. We found that secretion of pro-inflammatory factors such as TNF-α and IL-1β, and chemokines such as MIP-2 and RANTES by activated microglia was significantly inhibited when they were cultured in BMSC-CM. TNF-α and IL-1β have been proven to be toxic to surrounding tissues and neurons, while MIP-2 and RANTES are factors which participate in recruitment of many immune cells. Thus the ability of BMSCs to inhibit the production of pro-inflammatory factors indicates that BMSCs could have negative effects on diseases mediated by activated microglia. Anti-inflammatory factors such as IL-6 and IL-10 are generally considered to be potent immunosuppressive cytokines which regulate growth and differentiation of a variety of immune cells [34]–[36]. Our study shows that production of large amounts of IL-6 and IL-10 may contribute to the inhibitory effect of BMSCs on proliferation of microglia. The secretion of VEGF shows that BMSCs are probably related to the production of vessels in the CNS, which could explain why BMSCs facilitate wound healing in many types of injury. In addition to the secretion of neurotoxic mediators, phagocytosis of neurons by microglia is also considered to be one of the causes of neurodegenerative disorders [8]. Under normal circumstances, dead and dying neurons are quickly removed through microglial phagocytosis. However, under inflammatory conditions, the phagocytosis range of microglia is expanded, which usually leads to death of healthy neurons. Our data provide evidence that BMSCs inhibit phagocytosis by activated microglia, indicating that BMSCs can reduce neuronal death to a certain extent. High levels of CD68 (ED-1) expression are associated with activated microglia, while low levels of CD68 expression are associated with quiescent ramified microglia [37]. We proved in our experiment that BMSC-CM show an inhibitory effect on the surface expression of CD68 receptors, indicating that BMSCs could reduce the number of activated microglia. We further investigated possible reasons for the inhibitory effect of BMSCs. TUNEL staining clearly showed that BMSCs promote apoptosis of resting as well as activated microglia. This phenomenon demonstrates that BMSCs not only inhibit the proliferation of microglia but also promote their apoptosis. In the LPS-stimulated groups, apoptosis was reduced, possibly because, during inflammatory activation, macrophages (microglia are considered to be the CNS equivalent of macrophages) can use significant amounts of glycolytically-generated ATP to maintain high mitochondrial membrane potential and prevent apoptosis [38]. Many soluble factors have been shown to be related to the inhibitory effect of mesenchymal stem cells, and two of these – prostaglandin E2 (PGE2) and indoleamine 2,3-dioxygenase (IDO) – are the most important factors which have been reported to participate in MSC-mediated suppression of many immune cells [26], [39]. Inhibition of PGE2 synthesis has been shown to boost production of TNF-α and IFN-γ by dendritic cells co-cultured with MSCs, while inhibition of PGE2 and IDO simultaneously completely restored NK-cell proliferation [40]. In this study, we investigated whether NO was involved in the inhibitory effect of BMSCs on microglia. Other studies have reported that NO is one of the inhibitory factors, although the mechanisms remain vague [41]. High concentrations of NO have been shown to suppress Signal Transducer and Activator of Transcription (STAT)5 phosphorylation in T cells and to induce immune cell apoptosis in vitro [42]. We used the NO inhibitor S-methylisothiourea sulfate (SMT) to block secretion of NO by BMSCs, and found that cells cultured in this conditioned medium (with a low NO content) showed increased proliferation compared to cells cultured in conditioned medium containing normal amounts of NO. This result indicates that the inhibitory effect exerted by BMSCs involves production of soluble factors, and that NO is involved.

In summary, our present study shows that BMSCs have the ability to maintain the resting phenotype of microglia or to control microglial activation. By producing several regulatory factors, BMSCs not only inhibit microglial proliferation, but also markedly suppress major microglial functions, such as phagocytic activity and cytokine production. In addition, BMSCs promote microglial apoptosis via their production of soluble factors. We also demonstrate that NO participates in the inhibitory effect of BMSCs. The immunosuppressive capability of BMSCs indicates that they can protect neurons and glia from damage by weakening the destructive cascade reaction caused by acute or chronic microglial activation. Our in vitro experiments reveal that BMSCs may be a promising therapeutic option for some CNS diseases associated with microglial activation. However, it is obvious that further in vivo studies in animal models will be needed to confirm the relevance of our in vitro findings.

## Supporting Information

Figure S1 **Phenotype of rat bone marrow-derived mesenchymal stem cells and microglia.**
(TIF)
