# Supplementary figures and images for: Bone Marrow-Derived Mesenchymal Stem Cells Maintain the Resting Phenotype of Microglia and Inhibit Microglial Activation
Source: PLoS One. 2013 Dec 31;8(12):e84116. doi: 10.1371/journal.pone.0084116 (PMC3877190; doi:10.1371/journal.pone.0084116)

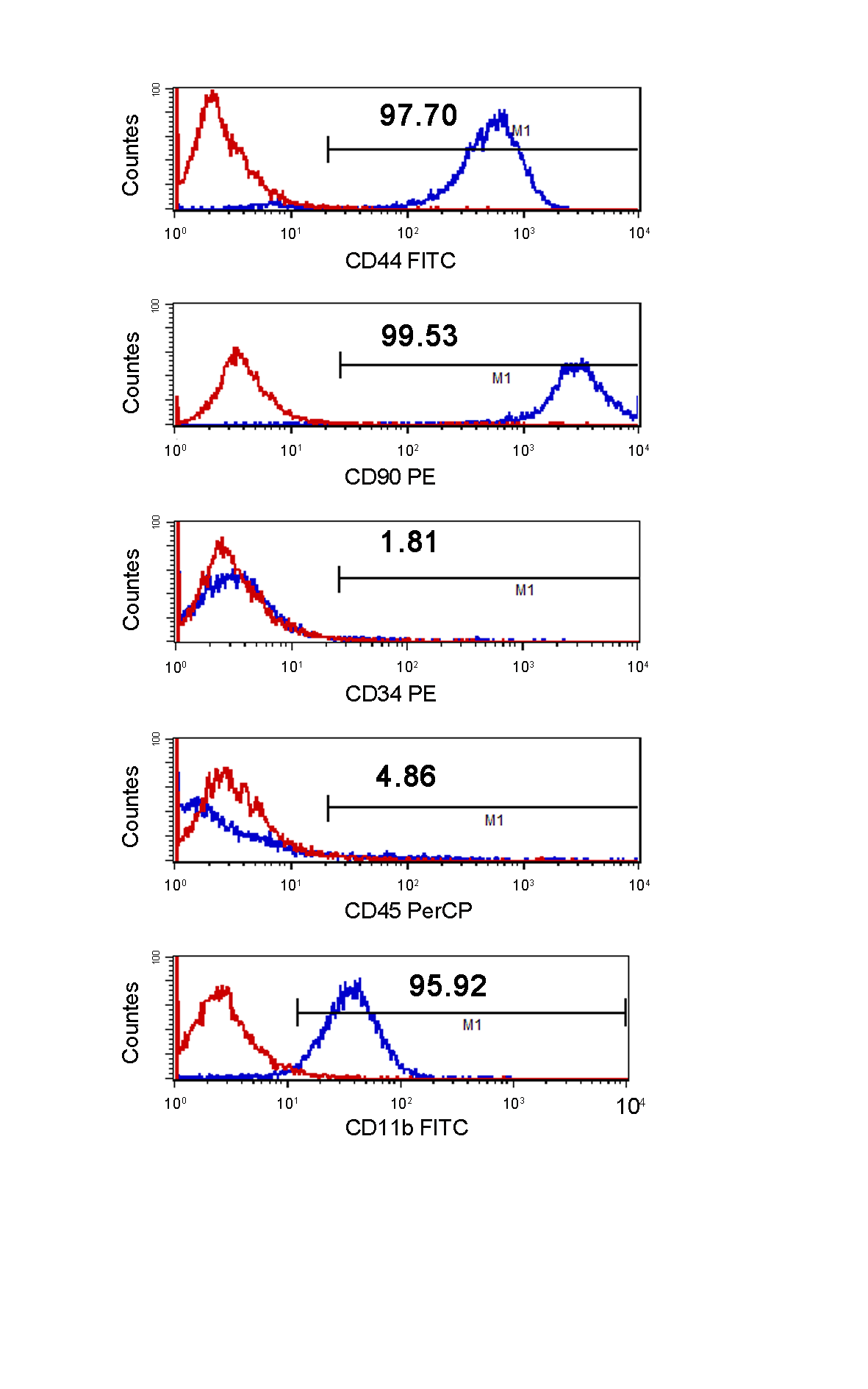

Supplement: Figure S1 — Phenotype of rat bone marrow-derived mesenchymal stem cells and microglia. (TIF) [file pone.0084116.s001.tif]
